# Guidelines from the Brazilian society of surgical oncology regarding indications and technical aspects of neck dissection in papillary, follicular, and medullary thyroid cancers

**DOI:** 10.20945/2359-3997000000607

**Published:** 2023-05-10

**Authors:** Terence Farias, Luiz Paulo Kowalski, Fernando Dias, Carlos S. Ritta Barreira, José Guilherme Vartanian, Marcos Roberto Tavares, Fernanda Vaisman, Denise Momesso, Alexandre Ferreira Oliveira, Rodrigo Nascimento Pinheiro, Heber Salvador de Castro Ribeiro

**Affiliations:** 1 Instituto Nacional de Câncer Ringgold Standard Institution Cabeça e Pescoço Rio de Janeiro RJ Brasil Instituto Nacional de Câncer, Ringgold Standard Institution, Cabeça e Pescoço, Rio de Janeiro, RJ, Brasil; 2 Pontifícia Universidade Católica do Rio de Janeiro Ringgold Standard Institution Pós-graduação em Cirurgia de Cabeça e Pescoço Rio de Janeiro RJ Brasil Pontifícia Universidade Católica do Rio de Janeiro, Ringgold Standard Institution, Pós-graduação em Cirurgia de Cabeça e Pescoço, Rio de Janeiro, RJ, Brasil; 3 Sociedade Brasileira de Cirurgia de Cabeça e Pescoço Ringgold Standard Institution São Paulo SP Brasil Sociedade Brasileira de Cirurgia de Cabeça e Pescoço, Ringgold Standard Institution, São Paulo, SP, Brasil; 4 Sociedade Brasileira de Cirurgia Oncológica Ringgold Standard Institution Rio de Janeiro RJ Brasil Sociedade Brasileira de Cirurgia Oncológica, Ringgold Standard Institution, Rio de Janeiro, RJ, Brasil; 5 A.C. Camargo Cancer Center Departamento de Cirurgia de Cabeça e Pescoço e Otorrinolaringologia São Paulo SP Brasil A.C.Camargo Cancer Center, Departamento de Cirurgia de Cabeça e Pescoço e Otorrinolaringologia, São Paulo, SP, Brasil; 6 Faculdade de Medicina da Universidade de São Paulo Ringgold Standard Institution Departamento de Cirurgia de Cabeça e Pescoço São Paulo SP Brasil Faculdade de Medicina da Universidade de São Paulo (FMUSP), Ringgold Standard Institution, Departamento de Cirurgia de Cabeça e Pescoço, São Paulo, SP, Brasil; 7 Cirurgia de Cabeça e Pescoço Hospital Dasa Brasília Brasília DF Brasil Hospital Dasa Brasília, Cirurgia de Cabeça e Pescoço, Brasília, DF, Brasil; 8 Instituto Nacional de Câncer Ringgold Standard Institution Seção de Cirurgia de Cabeça e Pescoço/Endocrinologia Rio de Janeiro RJ Brasil Instituto Nacional de Câncer, Ringgold Standard Institution, Seção de Cirurgia de Cabeça e Pescoço/Endocrinologia, Rio de Janeiro, RJ, Brasil; 9 Universidade Federal do Rio de Janeiro Rio de Janeiro RJ Brasil Universidade Federal do Rio de Janeiro, Endocrinologia, Rio de Janeiro, RJ, Brasil; 10 Universidade Federal de Juiz de Fora Ringgold Standard Institution Departamento de Oncologia Juiz de Fora MG Brasil Universidade Federal de Juiz de Fora, Ringgold Standard Institution, Departamento de Oncologia, Juiz de Fora, MG, Brasil; 11 Hospital de Base do Distrito Federal Ringgold Standard Institution Cirurgia Oncológica Brasília DF Brasil Hospital de Base do Distrito Federal, Ringgold Standard Institution, Cirurgia Oncológica, Brasília, DF, Brasil; 12 A.C. Camargo Cancer Center Ringgold Standard Institution Departamento de Cirurgia Abdominal São Paulo SP Brasil A.C.Camargo Cancer Center, Ringgold Standard Institution, Departamento de Cirurgia Abdominal, São Paulo, SP, Brasil

**Keywords:** Thyroid carcinoma, surgical treatment, papillary thyroid cancer, medullary thyroid cancer, follicular thyroid cancer, lymph node metastasis, elective neck dissection, therapeutic neck dissection, molecular tests, indications, guidelines

## Abstract

**Objective:**

The purpose of these guidelines is to provide specific recommendations for the surgical treatment of neck metastases in patients with papillary, follicular, and medullary thyroid carcinomas.

**Materials and methods:**

Recommendations were developed based on research of scientific articles (preferentially meta-analyses) and guidelines issued by international medical specialty societies. The American College of Physicians’ Guideline Grading System was used to determine the levels of evidence and grades of recommendations. The following questions were answered: A) Is elective neck dissection indicated in the treatment of papillary, follicular, and medullary thyroid carcinoma? B) When should central, lateral, and modified radical neck dissection be performed? C) Could molecular tests guide the extent of the neck dissection?

**Results/conclusion:**

Recommendation 1: Elective central neck dissection is not indicated in patients with cN0 well-differentiated thyroid carcinoma or in those with noninvasive T1 and T2 tumors but may be considered in T3-T4 tumors or in the presence of metastases in the lateral neck compartments. Recommendation 2: Elective central neck dissection is recommended in medullary thyroid carcinoma. Recommendation 3: Selective neck dissection of levels II–V should be indicated to treat neck metastases in papillary thyroid cancer, an approach that decreases the risk of recurrence and mortality. Recommendation 4: Compartmental neck dissection is indicated in the treatment of lymph node recurrence after elective or therapeutic neck dissection; “berry node picking” is not recommended. Recommendation 5: There are currently no recommendations regarding the use of molecular tests in guiding the extent of neck dissection in thyroid cancer.

## INTRODUCTION

Thyroid nodules are frequent in the adult population. The prevalence of palpable thyroid nodules is about 5% in women and 1% in men ( 1 , 2 ). However, high-resolution ultrasound can detect these nodules in up to 19%-68% of the cases, with this frequency being even higher in women and elderly individuals ( 3 , 4 ).

The main concern during an investigation of a thyroid nodule is to exclude or identify the presence of thyroid cancer, which is found in 7%-15% of the cases ( 5 , 6 ). Papillary (most frequent) and follicular carcinomas account for more than 90% of all cases of thyroid cancer ( 7 ).

With advances and widespread use of ultrasound and fine-needle aspiration biopsy, the incidence of thyroid cancer has been increasing. The frequency of tumors < 1 cm in new cases of thyroid cancer increased from 25% in 1988-1989 to 39% in 2008-2009. Additionally, the incidence of new cases tripled between 1975 and 2009, increasing from 4.9 to 14.3 per 100,000 individuals ( 8 ).

Notably, papillary thyroid carcinoma is often asymptomatic and incidentally detected in autopsy specimens ( 9 – 15 ). The highest prevalence ever reported of incidentally discovered papillary thyroid carcinomas in autopsies is 35.6% ( 9 ). In Brazil, the frequency of this finding is estimated at 1%-8% ( 16 ). Some authors in Japan even consider these incidentalomas to be normal findings; for them, papillary microcarcinomas (tumors < 5 mm) do not require treatment and should only be followed up ( 9 – 15 ). Indeed, according to some specialists, single papillary thyroid microcarcinomas < 10 mm without lymph node metastases or extrathyroidal extension can be managed without surgical treatment and with active surveillance alone ( 16 ). Another important finding in patients with papillary thyroid carcinoma is the presence of lymph node metastases; these can occur in 14%-64% of the cases overall and in up to 28% of the patients with tumors smaller than 1 cm ( 17 ).

The incidence of neck metastases is very low in follicular carcinomas, ranging in some studies from 2.1%-3.3% ( 18 – 19 ), but are more frequent in medullary thyroid carcinomas, ranging from 44%-81% ( 20 – 21 ).

Lymph node metastases are present in most patients with papillary thyroid carcinoma and are rare in those with follicular thyroid carcinoma ( 22 ). In papillary thyroid carcinoma, lymph node metastases have no prognostic significance in patients with low-risk disease but are predictive of poor survival in those with high-risk disease ( 23 ).

Neck metastases from papillary thyroid carcinomas occur most frequently to level VI. Central neck dissection includes the pre-laryngeal, pre-tracheal, and at least the paratracheal chains homolateral to the tumor. The dissection can include the retropharyngeal, retroesophageal, and para-laryngopharyngeal areas and the superior mediastinum and may extend inferiorly from the cricoid cartilage to the brachiocephalic vein. Intraoperative care should include the identification and preservation of the inferior laryngeal nerves (which must be dissected using an atraumatic technique) and inferior parathyroid glands. Intraoperative frozen section analysis is important to distinguish between parathyroid glands and lymph nodes, and if one or more parathyroids are accidentally resected, reimplantation should be performed ( 24 , 25 ).

A growing interest is to individualize the management of thyroid cancer based on risk stratification, establishing therapy and follow-up according to the estimated risk. The primary concern is to select the best therapeutic strategy to reduce morbidity and mortality and avoid overtreatment and its potential side effects. Thus, the surgical extent of the thyroidectomy and the neck dissection in patients with thyroid cancer must be carefully planned and individualized ( 26 , 27 ).

This document is the result of efforts by the Committee of Head and Neck Surgery of the Brazilian Society of Surgical Oncology to develop recommendations based on the current evidence available in the scientific literature regarding the indications and technical aspects of neck dissection in papillary, follicular, and medullary thyroid carcinomas, specifically:

- Indications for elective central compartment neck dissection in papillary, follicular, and medullary thyroid carcinomas.
- Indications for elective lateral neck dissection in papillary, follicular, and medullary thyroid carcinomas.
- Indications for therapeutic modified radical neck dissection in papillary, follicular, and medullary thyroid carcinomas.
- Management of lymph node recurrence after selective or therapeutic neck dissection in thyroid carcinomas.
- Recommendation regarding the use of molecular tests in guiding the extent of the neck dissection.

## MATERIALS AND METHODS

The development of the recommendations in the present guidelines was based on research of scientific articles published in English, including meta-analyses and guidelines from international societies such as the American Thyroid Association (ATA), the Japan Association of Endocrine Surgeons, and the European Thyroid Association (ETA). The research was carried out in the databases PubMed ( www.ncbi.nlm.nih.gov/pubmed ), SciELO ( www.scielo.org ), and LILACS (Bireme). The search was conducted manually or via the Internet.

The American College of Physicians’ Guideline Grading System was used as the grading system to determine the levels of evidence and grades of recommendations ( 28 ) ( Tables 1 and 2 ).

**Table 1:** Interpretation of the American College of Physicians’ Guideline Grading System ( 28 )

Grade of recommendation	Benefits versus risks and burdens	Implications
Strong recommendation	Benefits clearly outweigh risks and burden or vice versa	For patients, most would want the recommended course of action and only a small proportion would not; a person should request discussion if the intervention was not offered. For clinicians, most patients should receive the recommended course of action. For policymakers, the recommendation can be adopted as a policy in most situations.
Weak recommendation	Benefits closely balanced with risks and burden	For patients, most would want the recommended course of action but some would not – a decision may depend on an individual's circumstances. For clinicians, different choices will be appropriate for different patients, and a management decision consistent with a patient's values, preferences, and circumstances should be reached. For policymakers, policymaking will require substantial debate and involvement of many stakeholders.
Insufficient	Balance of benefits and risks cannot be determined	Decisions based on evidence from scientific studies cannot be made.

**Table 2:** Recommendation based on quality of evidence ( 28 )

Grade of recommendation/Quality of evidence	Methodological quality of supporting evidence	Interpretation
**Strong recommendation**		
High-quality evidence	Without important limitations or overwhelming evidence from observational studies	Can apply to most patients in most circumstances without reservation
Moderate-quality evidence	Important limitations (inconsistent results, methodological flaws, indirect, or imprecise) or exceptionally strong evidence from observational studies	Can apply to most patients in most circumstances without reservation
Low-quality evidence	Observational studies or case series	May change when higher quality evidence becomes available
**Weak recommendation**		
High-quality evidence	Without important limitations or overwhelming evidence from observational studies	Best action may differ depending on circumstances
Moderate-quality evidence	Important limitations (inconsistent results, methodological flaws, indirect, or imprecise) or exceptionally strong evidence from observational studies	Best action may differ depending on circumstances
Low-quality evidence	Observational studies or case series	Alternatives may be equally reasonable
Insufficient	Evidence is conflicting, poor quality, or lacking	Insufficient evidence to recommend for or against routinely providing the service

Based on the study of all the scientific articles surveyed, we propose the following questions to address the recommendations:

1. Is elective neck dissection recommended in the treatment of patients with papillary or follicular thyroid carcinoma?
2. Is elective neck dissection recommended in the treatment of patients with medullary thyroid carcinoma?
3. Is therapeutic posterior lateral neck dissection indicated in the presence of clinically detected metastases?
4. How should neck dissection be performed in recurrent disease after elective or therapeutic neck dissection in thyroid cancer?
5. Are molecular tests indicated to guide the extent of neck dissection?

## RESULTS

### Question 1:

#### Is elective central neck dissection recommended in the treatment of patients with cN0 well-differentiated thyroid carcinoma?

The 2020 revised guidelines of the Japan Association of Endocrine Surgeons ( 29 ) recommend elective central neck dissection in all cases of cN0 well-differentiated thyroid carcinoma. This recommendation was based on low scientific evidence but had good consensus-based approval. The recommendation was primarily based on a meta-analysis of 17 studies including 4,437 patients, in which elective central neck dissection along with total thyroidectomy was associated with a very low incidence of regional recurrence (0.66%; 95% confidence interval 0.49%-0.90%). Of note, the difference in recurrence rates with and without dissection was only 2.3%. This meta-analysis included three prospective and 14 retrospective studies, and the patients in both groups (with versus without dissection) had several differences. The authors further argued that the incidence of complications such as hypocalcemia was low, with odds ratios of 2.37 and 1.93 in patients who did and did not undergo dissection, respectively ( 30 ). They also recommended elective mediastinal dissection ( 31 ) on the basis that these lymph nodes are very difficult to identify before surgery using imaging tests ( 31 , 32 ); this recommendation is further supported by high complication rates in reoperations, *e.g.* , recurrent nerve injury ( 33 ).

The presence of lymph node metastases in papillary thyroid carcinoma has a low risk of recurrence (2.3%) and, according to one study ( 23 ) including 9,904 patients, has only a negative effect on survival when associated with other prognostic factors such as age above 45 years, distant metastases, and tumor size; indeed, the survival rates over 14 years have been described as 82% and 79% in the absence and presence of lymph node metastases, respectively.

Several studies have shown that elective central neck dissection does not improve survival rates or risk of recurrence and increases the risk of complications such as temporary hypocalcemia and dysphonia ( 34 – 39 ). These observations have also been shown in a Brazilian study by Ywata de Carvalho and cols. ( 40 ).

Elective resection of level VI lymph nodes in patients with cN0 neck staging identifies many patients with pN1 disease, but the direct long-term benefit of this approach is very small ( 40 – 42 ). Most groups only agree with this approach in high-risk cases (very old or very young patients, T3-T4 bulky tumors, multifocal aggressive disease, extrathyroidal extension, presence of lymph node metastases from levels II to V), in which staging influences decisions about adjuvant therapy. The use of currently available imaging methods prevents unnecessary elective neck dissections ( 34 , 38 , 41 – 44 ).

The presence of microscopic central lymph node disease is not a risk factor for macroscopic recurrence; this knowledge may prevent the misuse of radioiodine therapy ( 45 ). Following this same line of avoiding unnecessary treatment, Momesso and cols. suggest that radioiodine therapy should be avoided not only in patients with microscopic lymph node disease but also in those with primary tumors smaller than 2 cm without unfavorable factors (such as multifocal disease, extrathyroidal extension, positive lymph nodes or distant metastases). Therefore, radioiodine therapy should not be used indiscriminately ( 46 ). Vaisman & Tuttle have further emphasized that the management of these cases should be individualized and that stratification of risk groups is essential to help identify whether treatment should be implemented more or less aggressively ( 26 ).

**Recommendation 1: Elective central neck dissection is NOT recommended in the treatment of patients with cN0 papillary or follicular thyroid carcinoma.**

**Thyroidectomy without elective central neck dissection is appropriate for T1-T2 noninvasive tumors in patients with cN0 papillary thyroid carcinoma or follicular carcinoma.**

Strong recommendation/moderate-quality evidence.

**Elective central neck dissection in patients with cN0 papillary or follicular thyroid carcinomas should be considered only in those with advanced tumors (if papillary carcinoma), T3-T4 disease, in the presence of metastases in the lateral neck levels (II-V), or if this procedure is essential in planning adjuvant therapies.**

Weak recommendation/low-quality evidence.

### Question 2:

#### Is elective neck dissection recommended in medullary thyroid carcinoma?

The indication of total thyroidectomy with dissection of the central compartments depends on serum calcitonin levels and ultrasound findings. This is the standard treatment for sporadic or hereditary medullary thyroid carcinoma.

Patients with palpable tumors are at high risk for lymph node metastases. Nonetheless, several studies have reported that elective neck dissection is not associated with improved survival rates in medullary carcinoma. Kebebew and cols. and Grozinsky-Glasberg and cols. observed no differences in survival curves in studies including, respectively, 104 and 51 patients with medullary thyroid carcinoma ( 47 , 48 ). Of note, biochemical cure ( *i.e.* , negative calcitonin test) is more likely in patients who undergo elective neck dissection than in those who do not undergo this procedure.

The rates of levels VI or II-V neck metastases in patients without versus with neck dissection according to serum calcitonin values are, respectively, 12% versus 0% for calcitonin values 20-200 pg/mL, 43% versus 14% for values 200-2,000 pg/mL, 74% versus 44% for values 2,000-10,000 pg/mL, and 90% versus 80% for values above 10,000 pg/mL ( 49 ). Among patients with established metastases at level VI, the risk of metastases at levels II-V increases significantly, as does the incidence of contralateral metastases ( 50 ).

A question remains whether the extent of neck dissection influences prognosis in patients with medullary thyroid carcinoma. However, considering the rates of positive lymph nodes in these patients and the decrease in quality and survival time associated with recurrence, dissection of at least the central neck compartment (level VI) is indicated, an approach that is worthwhile for tumors > 5 mm or patients with calcitonin levels > 20 pg/mL. According to Wells ( 51 ), the encounter of metastases is very unlikely with preoperative calcitonin levels < 20 pg/mL. In the same publication, and based on expert opinion, the authors recommend contralateral neck dissection when calcitonin levels are > 200 pg/mL and preoperative imaging is positive in the ipsilateral neck compartment ( 51 ).

**Recommendation 2: Elective neck dissection of level VI (central) is indicated in all patients with medullary thyroid carcinoma.**

Moderate recommendation/moderate-quality evidence.

**Based on calcitonin levels: for levels between 20-200 pg/mL, dissection of the central compartment (level VI) is recommended; for levels above 200 pg/mL, bilateral modified radical neck dissection may be indicated when preoperative imaging is positive in the ipsilateral neck compartment.**

Weak recommendation/low-quality evidence.

### Question 3:

#### Can therapeutic modified radical neck dissection be performed?

A recent analysis of data from the US National Cancer Centers Network (NCCN) ( 52 ) has shown a small but significantly increased risk of death in patients with papillary thyroid cancer who are younger than 45 years. The study emphasized the importance of a more rigorous screening for neck metastases in this group of patients. Independent from other factors, the presence or absence of lymph node metastases has a small effect on the overall survival rates in patients with well-differentiated carcinoma. Still, there is a consensus that therapeutic neck dissection (levels II to VI) should be indicated in cases with neck metastases (cN1) ( 23 , 34 ).

Lymph node metastases occur most frequently in levels II-V (lateral and latero-posterior compartments), while level I is rarely affected ( 53 , 54 ). Neck dissection should be restricted to patients with clinically evident metastases or in whom metastases are observed on preoperative ultrasound and confirmed by cytological analysis of material collected by fine-needle aspiration and measurement of thyroglobulin in the aspirated wash. Also, in cases where suspicious lymph nodes are observed during surgery and lymph node metastasis is confirmed by frozen section examination, neck dissection (levels II-VI) can reduce the risk of recurrence and mortality ( 55 – 57 ).

Barbosa and cols. have shown that an elevated number of large metastatic lymph nodes in the lateral compartment and extracapsular leakage decrease the likelihood of an excellent response to initial treatment and are associated with neck lymph node recurrence or persistence and distant metastases ( 58 ).

The 2013 update of a Brazilian consensus of clinical endocrinologists on differentiated thyroid cancer recommended prophylactic dissection of the central compartment only when lymph node metastases are strongly suspected or confirmed on histopathology during surgery or in the presence of metastases in the lateral neck compartment. If metastatic lymph nodes are found in the lateral compartment but not in the central compartment, the central compartment (level VI) should also be dissected ( 59 ). Therefore, therapeutic neck dissection (sparing level I because of its low incidence of metastatic involvement) is mandatory when cervical metastasis is confirmed.

**Recommendation 3: Therapeutic lateral and central neck dissection (levels II-VI) must be performed in patients with papillary thyroid carcinoma in the presence of detected neck metastases confirmed by cytology, frozen section, or thyroglobulin measurement on aspirate wash. This approach decreases the risk of locoregional recurrence and mortality.**

- Strong recommendation/moderate-quality evidence.

### Question 4:

#### What is the surgical treatment for lymph node recurrence after elective or therapeutic neck dissection in thyroid cancer?

Some prognostic factors may influence the risk of neck lymph node recurrence or persistence, even in patients who have undergone more comprehensive types of elective neck dissections and in the hands of expert surgeons. These prognostic factors include age above 55 years, male sex, tumors with extrathyroidal extension, tumors larger than 3 cm, and lymph node characteristics such as number, size, location, and extranodal extension ( 46 , 58 , 60 – 62 )

Evidence from some observational studies suggests that disease recurrence affecting only a few lymph nodes can be managed conservatively (without surgery) under strict observation ( 63 , 64 ), but if the recurrence is large, invasive, or involves multiple lymph nodes, the treatment of choice is surgery ( 65 – 67 ).

Several factors must be considered in therapeutic decisions, such as the risks of a second surgery (since normal anatomic features may be distorted, especially when fibrosis is extensive) and damage to the parathyroid glands, inferior laryngeal nerves, carotid artery, internal jugular vein, and lymphatic duct, among others. Still, surgical resection is the treatment of choice for recurrent macroscopic lymph node disease ( 68 ).

The surgical indication should be based on clinical (structural disease) or radiological (ultrasound or computed tomography [CT] scanning) evidence of recurrence and confirmed by fine-needle aspiration (cytological evaluation or thyroglobulin measurement in the aspirate wash), instead of relying on increased serum thyroglobulin levels alone. Of note, confirmation of recurrence by cytopathology alone can be challenging ( 69 , 70 ).

Positron emission tomography with 2-deoxy-2-[fluorine-18]fluoro-D-glucose integrated with computed tomography (^18^F-FDG PET/CT) *or radioiodine s* ingle-photon emission computed tomography with integrated CT (RAI-SPECT/CT) may be used to improve the accuracy of the method for identification of recurrence. Lymph node tattooing with ultrasound-guided injection of activated charcoal can also be used ( 69 ).

Some authors advocate surgical treatment even in patients with few lymph nodes, regardless of risk level, in those with high-grade tumors, in cases with thyroglobulin doubling time (doubling of thyroglobulin values over time), in radioiodine-avid tumors (tumors with good radioactive iodine uptake) or those with good ^18^F-FDG-PET/CT uptake, and in the presence of molecular markers of aggressive behavior. The procedure known as “berry picking” (nodulectomy) is not recommended due to high recurrence rates, and compartmental surgery is indicated preferably ( 71 , 72 ).

Surgery, if indicated, should be safe and restricted to the affected compartment since the neck area with the recurrence has already been approached in previous neck dissection. The lateral compartment should be dissected if the recurrence is localized and restricted to this location and the compartment has not been dissected before. After compartmental dissection, baseline thyroglobulin levels decrease by 60%-90%, although a 30%-50% reduction has been described in some studies. Notably, the prediction of which patients will respond after salvage surgery is challenging. To minimize lesions to cervical structures, neurostimulators may be used to identify the inferior laryngeal nerve, and lymph nodes may be marked with technetium (^99m^Tc)-sestamibi for radioguided surgery or tattooed with charcoal ( 73 – 78 ).

**Recommendation 4: Surgical management of lymph node recurrence after elective or therapeutic neck dissection in thyroid cancer: central compartment neck dissection (level VI) should be performed, preferably sparing important anatomic structures. “Berry node picking” is not recommended. Standard therapeutic neck dissection is strongly recommended, if already not done, in cases of recurrence in the lateral compartments (levels II-V). The surgical approach should be restricted to the affected compartment.**

Strong recommendation/ moderate-quality evidence.

### Question 5:

#### Could molecular tests guide the extent of neck dissection?

A greater understanding of molecular signaling pathways could be used to stratify better patients who may benefit from prophylactic central neck dissection. Mitogen-activated protein kinase (MAPK) pathway mutations are well-known frequent driver mutations in papillary thyroid carcinomas. The most common genetic abnormality in papillary thyroid carcinomas is the BRAF V600E mutation, which has a reported frequency of 40%-70% ( 79 ). In a retrospective review by Tufano and cols., BRAF V600E mutations were reported in 75% of 120 patients who underwent central neck dissection due to disease recurrence in this compartment ( 80 ). Additionally, Howell and cols. have shown that, among preoperative clinical endpoints commonly used to determine prophylactic central neck dissection, only BRAF V600E mutation was an independent predictor of the presence of metastases in a cohort of 156 patients ( 81 ).

A study from four institutions in the United States ( 82 ) has shown that the detection of BRAF V600E mutation was not superior to intraoperative examination in predicting the presence of metastatic lymph nodes in the central compartment. Recently, patients carrying a BRAF V600E mutation combined with a telomerase reverse transcriptase (TERT) promoter mutation have been shown to present a significantly shorter progression-free survival, clearly suggesting a more aggressive tumor biology in these cases. Still, based on current evidence, no definitive molecular marker – not even the BRAF V600E mutation – can reliably predict the presence of metastases in the central compartment, and no data have shown that prophylactic resection changes the outcome in patients carrying these mutations ( 83 ).

**Recommendation 5: The use of molecular tests to guide the extent of neck dissection in patients with thyroid cancer is not recommended.**

Strong recommendation/moderate-quality evidence.
